# Prognostic factors in patients with advanced differentiated thyroid cancer treated with multikinase inhibitors – a single Brazilian center experience

**DOI:** 10.20945/2359-3997000000364

**Published:** 2021-04-29

**Authors:** Natalia Treistman, Gabriela Maia Nobre, Mariana Yoshii Tramontin, Gabriel Madeira Werberich da Silva, Daniel Herchenhorn, Luiz Henrique de Lima Araujo, Fernanda Accioly de Andrade, Rossana Corbo, Daniel Bulzico, Fernanda Vaisman

**Affiliations:** 1 Instituto Nacional do Câncer Departamento de Medicina Rio de Janeiro RJ Brasil Departamento de Medicina, Serviço de Endocrinologia, Instituto Nacional do Câncer (Inca), Rio de Janeiro, RJ, Brasil.; 2 Universidade Federal do Rio de Janeiro Hospital Universitário Clementino Fraga Filho Departamento de Medicina Rio de Janeiro RJ Brasil Departamento de Medicina, Serviço de Endocrinologia, Hospital Universitário Clementino Fraga Filho, Universidade Federal do Rio de Janeiro, Rio de Janeiro, RJ, Brasil.; 3 Instituto Nacional do Câncer Departamento de Medicina Rio de Janeiro RJ Brasil Departamento de Medicina, Serviço de Oncologia, Instituto Nacional do Câncer (Inca), Rio de Janeiro, RJ, Brasil.; 4 Instituto D'Or de Pesquisa e Educação Grupo de Oncologia D'Or Rio de Janeiro RJ Brasil Grupo de Oncologia D'Or, Instituto D'Or de Pesquisa e Educação (IDOR), Rio de Janeiro, RJ, Brasil.

**Keywords:** Differentiated thyroid cancer, radioactive iodine refractory, multikinase inhibitor therapy, real-world data

## Abstract

**Objective:**

The aim of this study was to describe the real-world experience multikinase inhibitors (MKI) in the treatment advanced differentiated thyroid carcinoma (DTC) refractory to radioactive iodine (RAIR) therapy.

**Subjects and methods:**

We reviewed the records of all patients with MKI-treated DTC from 2010 to 2018. Progression free survival (PFS), response rates (RR) and adverse events (AE) profiles were assessed. Clinical parameters were compared between groups with different outcomes (disease progression and death) to identify possible prognostic factors and benefit from treatment.

**Results:**

Forty-four patients received MKI for progressive RAIR DTC. Median PFS was 24 months (10.2-37.7) and median overall survival (OS) was 31 months. Best overall response was complete response in one patient (4.5%), partial response in nine (20.4%), stable disease in twenty-two (50%), and progressive disease (PD) in twelve (27.3%). Seventy-two point 7 percent patients had clinical benefit and AE were mild in most cases (82.7%). Progressive patients were more likely to have FDG positive target lesion than those who did not progress (p = 0.033) and higher maximum SUV on target lesions (p = 0.042). Presence of lung-only metastasis and lower thyroglobulin (Tg) during treatment was associated with stable disease (p = 0.015 and 0,049, respectively). Patients with shorter survival had larger primary tumor size (p = 0.015) and higher maximum SUV on target lesions (p = 0.023).

**Conclusion:**

Our findings demonstrate safety and effectiveness of MKI in patients with advanced RAIR DTC. We were able to identify as possible prognostic markers of better outcomes: absence of FDG uptake on target lesions, lower maximum SUV on PET-CT, presence of lung-only metastasis and lower Tg during treatment.

## INTRODUCTION

Differentiated thyroid carcinoma (DTC) is the most common endocrine malignancy and its incidence has been rising worldwide ( 1 ). In Brazil, estimates for 2018-2019 indicate 9610 new cases ( 2 ).

In general, DTC has excellent prognosis and over 98% 5-year overall survival (OS) rates. Despite representing about 3% of new cancer cases in the US, it is responsible for less than 0.3% of cancer-related deaths ( 3 ). However, there is a small group of patients that can have a worse prognosis and need for additional therapy besides surgery and radioactive iodine (RAI). It is also known that patients with metastatic disease sensitive to RAI have better outcome than those who are not ( 4 ).

For patients with advanced and metastatic disease who are refractory to RAI (RAIR), therapeutic options are limited and overall response rates (RR) are also modest. Historically, it is known that DTC has poor response to cytotoxic chemotherapy ( 5 , 6 ).

Over the last 15 years, knowledge on molecular mechanisms involved in DTC carcinogenesis and progression has evolved substantially, and with that new therapeutic possibilities were discovered ( 7 – 9 ). Multikinase inhibitors (MKI) were first used to treat hematologic malignancies, liver and renal cancers and were more recently approved for progressive RAIR DTC. In Brazil, the two approved MKI for RAIR DTC are sorafenib and lenvatinib, but those agents are not widely available for the public health system ( 10 ).

The experience of MKIs in DTC is still growing in many settings. Since the release of prospective controlled studies, many authors have published their experience with these agents in real-life scenarios and reported important differences in this context ( 11 – 31 ). However, Brazilian experience is still limited and there is no large DTC experience reported.

The aims of this study were to analyze and describe the experience of a Brazilian referral center in oncology with the use of MKI in the treatment of patients with advanced RAIR DTC and to identify predictive and prognostic factors associated with treatment.

## SUBJECTS AND METHODS

We retrospectively reviewed medical records of all MKI-treated DTC patients at a single center – National Cancer Institute (Inca) –, Rio de Janeiro, Brazil, from December 2010 to November 2018.

Inclusion criteria were patients > 18 years diagnosed with advanced DTC treated with MKI. For our analysis, we included all patients, even those with short-term treatments (less than 3 months before progression, treatment discontinuation or death).

Patients younger than 18 years old, medullary thyroid carcinoma or anaplastic thyroid carcinoma, or patients with DTC not treated with MKI were excluded.

The following demographic and clinical data from all subjects included in the analysis were collected: gender, age at diagnosis, tumor histology, number of RAI treatments, cumulative RAI activity, whole body survey (WBS) results after therapeutic RAI, criteria used to determine RAIR disease, tumor staging, metastatic lesion sites, target lesion size and site, other systemic or localized therapies performed, adequate TSH suppression prior to MKI, date and dosage of MKI treatment initiation, dosage modification when it occurred, temporary discontinuation of treatment, adverse events (AE) and its degree when present, treatment discontinuation date and motive, anti-thyroglobulin (ATg) antibody levels and serum thyroglobulin (Tg) before treatment, lower ATg and Tg during treatment, imaging studies during follow-up and structural response, time of last visit during follow-up, date of death. Tumor stage was classified according to AJCC/TNM 8^th^ edition ( 32 ).

Criteria used to determine RAIR disease was defined using the American Thyroid Association guidelines' definition ( 6 , 33 ).

Patients who had clinical and radiological progressive RAIR disease were evaluated for MKI therapy. Therapy was initiated in those with symptomatic progression or with disseminated disease not manageable with localized therapy. In general, therapy was not indicated in asymptomatic patients with target lesions smaller than 2 cm in the largest diameter. To be eligible for treatment, patients must have had documented disease progression within 14 months.

At our institution, patients on MKI therapy are followed by a multidisciplinary team, including endocrinology, oncology, dermatology, and nurses. Depending on the case, voice therapist, clinical pain specialist and others may be involved. Initial treatment with MKI requires shorter clinical reevaluations (every 15 to 30 days) for dose adjustments and management of possible AE, and then clinical and laboratory reassessment is performed every 2 to 3 months. The severity of AE is graded according to the National Cancer Institute Common Terminology Criteria for Adverse Events, version 4.0. Imaging and structural response studies were evaluated according to a certified radiologist (PD being defined as at least 20% increase in measurements and partial response [PR] as decrease in at least 30% of target lesions).

PFS was defined as the time between initiation of MKI therapy and the first documentation of radiological disease progression, death or loss of follow-up. OS was defined as the time between MKI therapy initiation and death, loss of follow-up or last clinical visit.

Functional sensitivity of the serum Tg assay varied over the years. From 2001 to 2010, serum Tg was quantified by immunometric assay (Immulite) with functional sensitivity of 0.2 ng/mL, and from 2010 to the present functional sensitivity dropped to 0.1 ng/mL (Elecsys Tg II test).

Dosages of ATg, TSH and free T4 are currently performed with electrochemiluminescence immunoassays. Functional sensitivity of ATg assay is currently 10.0 IU/mL (Elecsys Anti-Tg test). TSH Functional sensitivity is 0.005 μIU/mL (Elecsys TSH test) and free T4 is 0.5 pmol/L (Elecsys FT4 II test).

### Ethical guidelines

This work has been approved by Inca's ethics research committee under the number 40788815.0.1001.0065.

Statistical analysis was performed using SPSS version 20.0 (SPSS Inc., Chicago, IL, USA). Continuous variables were described as means and medians, categorical variables, presented as numbers and percentages. Parametric variables were evaluated with chi-square and Student's t test. Nonparametric variables were evaluated by the Mann-Whitney U test. Survival curves were performed by the Kaplan-Meier method, and the log-rank test was used to determine statistical significance. The confidence interval is 95% and p value was considered statistically significant < 0.05.

## RESULTS

In total, 44 patients were included in the analysis and their medical records were reviewed. Baseline characteristics are described in Table 1 .

**Table 1:** Baseline characteristics

	N = 44	%
Age (years)	60.8 (34-79)	
Sex F:M	27:17	61.4: 38.6
Size (cm)	4.6 (1.1-11.5)	-
Histology		
Papillary	31	70.5
Follicular	12	27.2
Poorly differentiated	1	2.3
Follicular variant papillary	6	13.6
Hürthle Cell	3	6.8
Insular	3	6.8
Tall cell	1	2.3
8th edition AJCC		
Tx	21	47.8
T1a	0	0
T1b	0	0
T2	6	13.6
T3a	4	9.0
T3b	1	2.3
T4a	7	15.9
T4b	5	11.4
Nx	31	70.5
N0	1	2.3
N1a	4	9.0
N1b	8	18.2
M1	23	52.2
At least one RAI treatment	41	93.2
RAI activity (mCi)	422.5 (150-1000)	-
Symptoms before MKI	20	45.5
Time from diagnosis to MKI (years)	68.7 (0.3-210.1)	-
Additional therapy besides MKI		
External beam radiation	27	61.4
Chemotherapy	3	6.8
Embolization	3	6.8
Zoledronate	6	13.6
Final status		
Stable disease	11	25
Complete response	1	2.3
Progression	7	15.9
Disease related death	25	56.8
PFS on MKI (months)	24 (10.2-37.7)	-
OS after MKI (months)	31 (17.7-44-.2)	-
Follow up (months)	99.6 (12.5-236.3)	-

MKI: multikinase inhibitors; PFS: progression free survival; OS: overall survival; RAI: radioiodine.

Twenty-seven (61.4%) patients were female and 17 (38.6%) male. Mean age at diagnosis was 60.8 and mean age at the beginning of MKI treatment 69.3 years. Regarding tumor histology, 31 patients (70.5%) had papillary thyroid carcinoma (PTC), 12 (27.2%) had follicular thyroid carcinoma (FTC) and 1 patient (2.3%) had poorly differentiated thyroid carcinoma (PDTC). Among PTC patients, 6 had follicular variant papillary, 3 patients had insular variant, 1 tall cell (13.6%, 6.8% and 2.3%, respectively). Regarding FTC, 3 patients had oncotic variant (6.8%).

Twenty-three patients (52.2%) already had distant metastases at diagnosis. Forty-one patients were treated with RAI. They received median cumulative activity of 422.5 mCi (150-1,000). Three patients were not treated with RAI due to unresectable disease and large remaining volume of thyroid tissue.

Criteria used to determine RAIR disease was negative WBS in 40.5%, PD less than 16 months after RAI treatment in 27.5% and cumulative RAI activity over 600mCi without remission of disease in 27.5% of cases.

Regarding metastatic lesions sites, 40 patients (91%) had pulmonary metastasis, 9 of those (20.45%) had exclusively pulmonary metastasis. Sixteen patients (36.3%) had bone metastasis and 10 (22.7%) patients had metastasis in other sites, including liver, pancreas and the pituitary gland. Target lesions were pulmonary in 27 cases (61.4%), cervical masses or lymph nodes in 8 cases (18.2%), bone metastasis in 5 cases (11.4%) and 4 cases had target lesions located in other areas. Average target lesions size was 3.1 cm. All patients were evaluated with PET-CT, except one.

Median time between DTC diagnosis and initiation of MKI therapy was 68.7 months (0.3-210.1). Forty patients used sorafenib and 4 patients used vandetanib, all as first line treatment. Average initial dose of sorafenib was 760 mg/d, with 36 patients starting 800 mg. Initial dose for vandetanib varied between 100 mg/d and 300 mg/d.

Prior to MKI therapy 45.5% of patients had adequate TSH suppression (TSH < 0.1 μIU/mL at least 9 of 12 previous months). Mean serum Tg before MKI was 6,469.4 ng/mL and mean ATg titers 197.4 IU/mL, mean lowest Tg during MKI treatment and lowest ATg during MKI treatment were 804.3 ng/mL and 57.9 IU/mL, respectively.

Regarding best response during treatment with MKI, 9 (20.4%) patients had PR, 22 patients (50%) had SD and 12 cases (27.3%) had PD as best response during treatment as shown in Table 2 . One patient presented complete response (CR) criteria and this case will be further discussed later. Overall, 72.7% patients had clinical benefit from MKI treatment, defined as the sum of CR, PR and SD. Twenty patients had symptomatic disease before starting MKI. 13 of them (65%) reported clinical improvement of symptoms some time during treatment.

**Table 2:** Response to therapy RECIST 1.1

N = 44	Best response to MKI therapy
Complete response	1 (2.3%)
Partial response	9 (20.4%)
Stable disease	22 (50%)
Clinical benefit	32 (72.7%)
Disease progression	12 (27.3%)

MKI: multikinase inhibitors.

**Figure 1:**
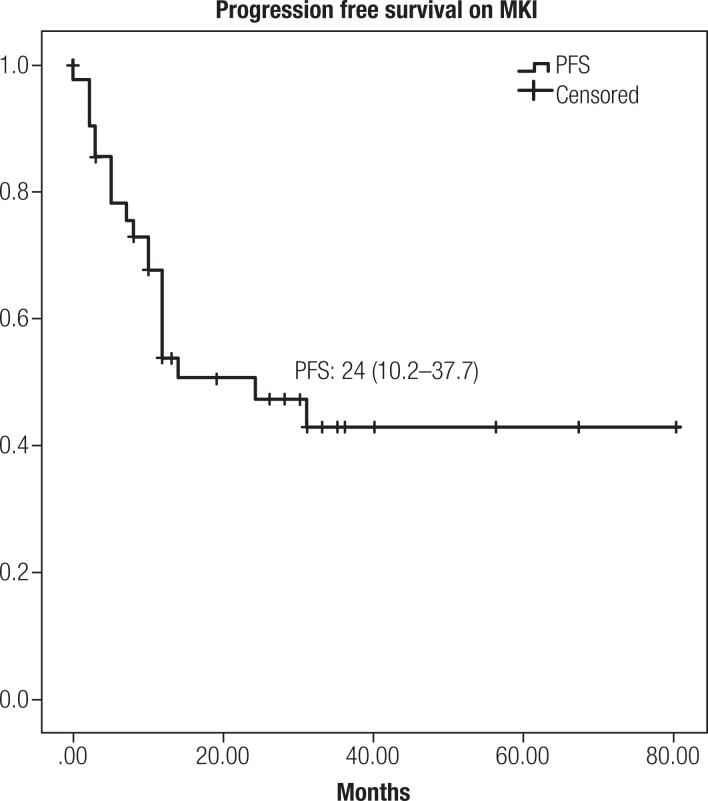
Progression free survival during MKI (in months).

**Figure 2:**
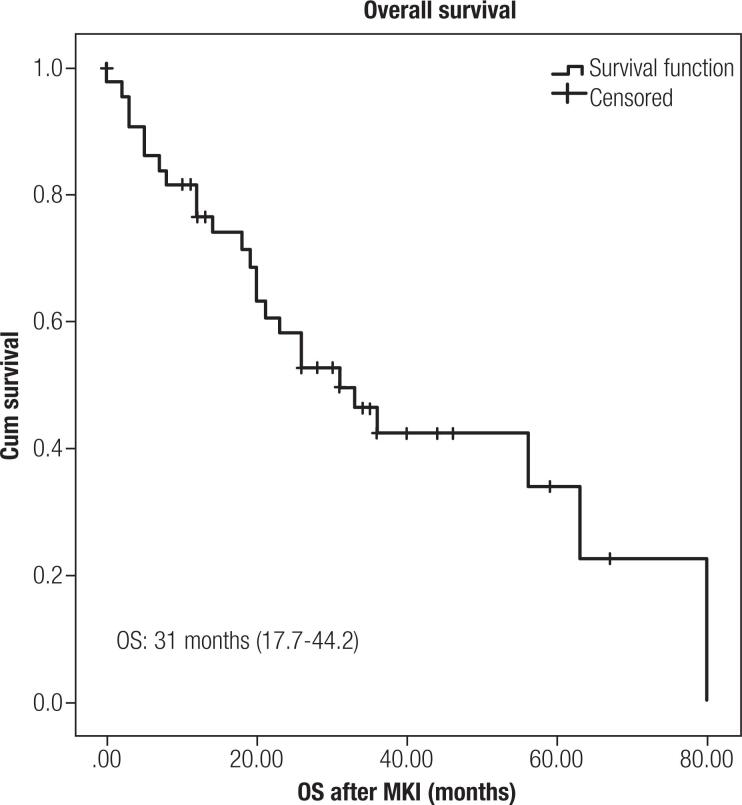
Overall survival after MKI (in months).

Median PFS was 24 months (10.2-37.7) ( Figure 1 ) and median OS was 31 months (17.7-44.2) ( Figure 2 ). Median follow-up of 99.6 (12.5-236.3) months. Duration of response for the entire cohort was 12 months (0.5-800, for PR 12 months (8-35), for SD 31 (5-80) and for PD 9 (0-31 months).

Forty-three patients presented AE during treatment, only 1 patient had no AE reported (results in Table 3 ). In total, 168 AE were described, 139 (82.7%) mild (grades 1 or 2) and 29 (17.3%) grade 3 or 4. 21 patients (47.7%) required temporary discontinuation of medication due to AE. Twenty-two patients (50%) required dose reduction and 11 cases (25%) had the drug suspended due to AE. One patient had cutaneous neoplasia secondary to MKI use.

**Table 3:** Adverse events during MKI treatment

	Any grade	G1-G2 (%)	G3-G4 (%)
Hand-foot syndrome	30	22 (50)	8 (18.2)
Diarrhea	31	25 (56.8)	6 (13.6)
Fatigue	31	29 (65.9)	2 (4.6)
Hypertension	5	4 (9.0)	1 (2.3)
Alopecia	11	9 (20.4)	3 (6.8)
Anorexia	5	5 (11.3)	0
Weight loss	8	8 (18.2)	0
Nausea	9	8 (18.2)	1 (2.3)
Rash	7	5 (11.3)	2 (4.6)
Hematologic toxicity	1	1 (2.3)	0
Pruritus	1	1 (2.3)	0
Secondary neoplasia	1	0	1 (2.3)

MKI: multikinase inhibitors.

We also analyzed and compared data from patients who had PD on MKI with those who did not have PD while using MKI. These analyses are presented in Table 4 . We found no difference between the group that progressed and those that did not progress regarding age, gender, symptoms at the beginning of MKI treatment, number of metastatic sites, number of AE or average RAI activity.

**Table 4:** Progression on MKI

		Progression ( 22 )	No progression ( 22 )	p-value
Age (years)		59	66	0.561
Sex (F)		50%	72.7%	0.215
Primary tumor size (cm)		4.5	3.7	0.057
Number of metastatic sites				0.03
	1	13.6%	45.5%	
	2	45.5%	31,8%	
	3	22.7%	18.2%	
	4	18.2%	4.5%	
Pulmonary metastasis only		4.5%	36.36%	**0.02**
Max. SUV - PET + Target lesion		14.62	11.0	**0.042**
PET + Target lesion		100%	85.7%	**0.033**
Lowest Tg during MKI		664.9	165.5	**0.049**
Symptomatic disease		54.5%	36.4%	0.364
Number of AE		3.0	4.0	0.213
Mean RAI activity		365.79	473.81	0.057

MKI: multikinase inhibitors; Tg: thyroglobulin; RAI: radioiodine, AE: adverse events.

On uni-variate analysis patients who had PD on MKI were more likely to have FDG uptake on target lesions on PET-CT when compared to patients who did not progress (p = 0.033) and higher maximum SUV on PET-CT on target lesions (p = 0.042). Presence of lung-only metastasis was associated with no PD (p=0.021). Patients who did not progress had on average lower Tg during treatment when compared to patients who progressed (p = 0.049), however there was no statistically significant correlation with initial Tg.

We performed analysis comparing patients who died during or after MKI treatment and survivors, as shown in Table 5 .

**Table 5:** Disease related death

		Deaths ( 25 )	Survivors ( 19 )	p-value
Age		61.16	60.42	0.649
Sex (F)		72%	47.4%	0.125
Primary tumor size		5.47	3.6	**0.035**
Number of metastatic sites				
	1	20%	42.2%	**0.02**
	2	44%	31.5%	
	3	16%	26.3%	
	4	20%	0%	
PET + target lesion		96%	84.2%	**0.023**
Lowest Tg during MKI		914.11	701.75	0.088
Symptomatic disease		56%	31.6%	0.135
PD target lesion vs. Non target lesion		64.7%	75%	0.689
Number of AE		3.6	4.1	0.530
Mean RAI activity		452.27	386.11	0.407

MKI: multikinase inhibitors; Tg: thyroglobulin; RAI: radioiodine, AE: adverse events.

Patients who died had larger primary tumor size (p = 0.035), more frequently had more than one site of distant metastasis (p = 0.002) and higher incidence of glucose uptake on target lesions on PET-CT (p = 0.023).

## DISCUSSION

In this study we describe a retrospective cohort of patients with progressive unresectable DTC RAIR, treated with MKI for a median period of 99.6 months in a public referral center in Rio de Janeiro. This larger Brazilian experience showed that, in a real-world study, median PFS was 24 months (10.2-37.7) and OS was 31 months (17.7-44.2), with frequent but manageable adverse events in properly selected patients.

Despite the favorable results of previous phase III studies, there are still many unresolved questions regarding the clinical management of patients treated with MKI treated RAIR DTC. Chief among them is how such results are converted to a real-life scenario practice. Several groups have begun to describe their experience with treating DTC using MKI and its feasibility in many different countries, continents, and contexts ( 13 – 31 , 34 , 35 ). Findings of previous colleagues as well as our results are summarized in Table 6 . Our study represents a large single center cohort treated with MKI, with long follow-up, being one of the few cohorts in South American and the first with Brazilian population.

**Table 6:** Review of world real-life experience in use of MKI in DTC

Country	Year	Authors	Number of centers	Drugs	Number of subjects	1st line MKI or more	Median PFS (months)	Prognostic Factors
United States	2010	Cabanillas and cols.	Single center	Sorafenib Sunitinib	15 DTC	1^st^ line or more	19	Yes: Log Tg
Italy	2013	Marotta and cols.	Single center	Sorafenib	17	1^st^ line	9	Yes: Tg levels and Tg response to treatment, baseline FDG-PET
France	2014	Massicotte and cols.	Multicenter	Sorafenib Sunitinib Vandetanib	45 DTC (17 MTC)	1^st^ line or more	7.0 (1st line DTC)	No
Turkey	2015	Benekli and cols.	Unclear (Turkish Ministry of Health database)	Sorafenib	14 DTC (16 MTC)	Unclear	21.3 (DTC group)	No
France	2017	Berdelou and cols.	Multicenter	Lenvatinib	75	1^st^ line or more	10	No
Spain	2018	Molina-Vega and cols.	Single center	Sorafenib Lenvatinib Axitinib	17	1^st^ line or more	18	No
Korea	2018	Mijin Kim and cols.	Multicenter	Sorafenib	98	1^st^ line	9.7	Yes: Symptoms, lung-only metastasis, daily maintenance dose, Tg reduction
Switzerland*	2018	Balmelli and cols.	Multicenter	Lenvatinib	13	1^st^ line or more	7.2	Yes: Tg levels (with radiologic response)
Japan	2018	Sugino	Single center	Lenvatinib	29	1^st^ line or more	24.3	Symptom
Korea	2019	Kim and cols.	Multicenter	Sorafenib	85	1^st^ line or more	14.4	Yes: Small tumor size, long doubling time
Japan	2019	Suzuki and cols.	Single center	Lenvatinib	26	1^st^ line or more	2 year-PFS= 58.4%	Yes: Baseline tumor size and symptoms
Japan	2019	Yamazaki and cols.	Single center	Lenvatinib	36	1^st^ line or more	Full Dose: 696 days Low Dose: not reached	No
Korea	2019	Lee and cols.	Multicenter (11)	Lenvatinib	67	1^st^ line or more	5.1	Yes: Rapidly PD with shorter initial tumor doubling time
Italy	2019	Locati and cols.	Multicenter (16)	Lenvatinib	94	1^st^ line or more	10.8	No
Argentina	2019	Jerkovich and cols.	Single center	Sorafenib Lenvatinib	22	1^st^ line or more	31.5 (16.5 −1^st^ line only)	No
Japan	2019	Iwasaki and cols.	Multicenter	Sorafenib Lenvatinib	56	1^st^ line	Median treatment duration: Sorafenib 5.1 Lenvatinib 14.1	Yes: Pulmonary metastasis as target lesion
Portugal	2019	Santos and cols.	Single center	Sorafenib Sunitinib	28	1^st^ line or more	10.8 (1^st^ line sorafenib)	No
China	2020	Cheng and cols.	Single center	Sorafenib	72	1^st^ line	17.6	Yes: Hand-foot syndrome, Well DTC, ECOG PS ≤ 2, biochemically nonineffective response, lung-only metastasis, and absence of bone metastasis
Argentina	2020	Jerkovich and cols.	Multicenter (02)	Lenvatinib	22	1^st^ line or more	13.7	No
Netherlands	2020	Aydermirli and cols.	Multicenter (03)	Lenvatinib	39	1^st^ line or more	9.7	No
Japan	2020	Masaki and cols.	Single center	Lenvatinib	42	1^st^ line or more	13.8	No
Brazil	2020	Treistman and cols.	Single center	Sorafenib	44	1^st^ line	24	FDG uptake on target lesions on PET-CT, higher SUV presence of lung-only metastasis and lower Tg during treatment

TTg: thyroglobulin; DTC: differentiated thyroid cancer; MTC: medullary thyroid cancer; PFS: progression free survival; OS: overall survival; RAI: radioiodine.

Regarding survival outcomes, our findings are slightly different from previous phase III studies but consistent with other groups reports of real-world experience, such as Cabanillas and cols. with 19 months PFS in a North American cohort, Benekli and cols. with 21.3 months PFS in Turkish population, Molina-Vega and cols. with 18 months PFS in a Spanish cohort, Sugino and cols. 24.3 months in a Japanese cohort, and Jerkovich and cols. with 31.5 months PFS in an Argentinian cohort ( 11 – 13 , 18 , 25 , 26 , 29 ).

Clinical trials DECISION and SELECT have previously showed PFS of 10.8 months and 18.3 months, respectively, an improvement when compared to their placebo groups, respectively, 5.8 and 3.6 months ( 11 , 12 ). Although not directly comparable, considering all our subjects presented documented PD within 14 months prior to MKI initiation, we believe our finding of median 24 months PFS demonstrates the usefulness of MKI treatment to prevent disease progression.

Most of our patients experienced clinical benefit of treatment. 50% of them had SD, 20.4% PR and one presented CR. This patient was started on MKI after presenting a rapidly progressive unresectable endotracheal lesion that can no longer be seen on cross sectional images after 28 months of sorafenib. Our 72.7% clinical benefit was similar to Marotta and cols. 71% (30% PR and 41% SD) and Iwasaki and cols.'s 75.0% disease control rate (PR plus SD) ( 14 , 34 ).

Three of our patients did not receive RAI due to unresectable disease and large remaining volume of thyroid tissue, similar cases have also been reported in previous cohorts. Santos and cols., Berdelou and cols. as well as Locati and cols. also described in each report patients that did not undergo thyroid surgery before starting MKI therapy due to unresectable tumors ( 16 , 22 , 30 ). Those patients would not be eligible for previous MKI trials, however in our experience, two of those three patients had clinical benefit of MKI treatment (one PR and one SD).

When we compared groups divided by outcomes (PD on MKI versus no PD) we found no difference regarding age, number of AE or average RAI activity. We also found no difference regarding symptoms at the beginning of MKI treatment and disease progression as some groups have previously reported. Both Suzuki and cols. and Sugino and cols. have reported that tumor-related symptom were prognostic factors for both poorer PFS and OS in Japanese cohorts ( 29 , 36 ). Kim and cols. also found such association in a multicenter Korean cohort ( 20 ). This difference in our results could be explained due to sample size or perhaps different studied population. Even though symptomatic disease did not correlate with PD or death outcomes in our study, 65% of patients who had symptomatic disease before starting MKI reported clinical improvement of symptoms some time during treatment. Berdelou and cols. also described that 52% of their 44 patients with initial symptoms related to DTC had clinical improvement of symptoms ( 16 ).

Another interesting finding was that presence of lung-only metastasis was associated with no PD (p = 0.021) and that patients who did not progress had on average lower Tg during treatment when compared to patients who progressed (p = 0.049). Kim and cols. also described association between lung-only metastasis and PFS, Cheng and cols. also reported that better PFS and OS were found in patients with lung-only metastasis ( 17 , 20 ).

Several authors also found correlations between Tg levels and response to MKI. First, Cabanillas and cols. reported that lower Log Tg was associated to better radiological response ( 13 ). Marotta and cols. described that baseline Tg levels were significantly higher in patients who showed disease progression, as well as correlation between baseline Tg and PFS ( 14 ). This group also reported that the decrease in serum Tg levels was significantly greater in patients who achieved clinical benefit. In Balmelli and cols.'s report decrease in Tg levels correlated with radiologic response in 6 evaluated patients ( 31 ). In Korean population, 60% Tg reduction was associated with better PFS, and more recently Cheng and cols. biochemically response (decrease Tg, stable Tg or increases of under 25%) independently predicted PFS and OS ( 17 , 20 ).

As of major interest, risk factors for cancer-specific mortality was deeply explored. Our group found no difference regarding age, gender, PD site, number of AEs, symptoms at the beginning of MKI therapy or mean RAI activity. Patients who died had larger primary tumor size (p = 0.035) and higher incidence of glucose uptake on target lesions on PET-CT (p = 0.023). Our group also showed that patients who evolved with PD had a higher incidence of FDG uptake on target lesions on PET-CT when compared to patients who did not progress (p = 0.033) and higher maximum SUV on PET-CT on target lesions (p = 0.042). Association between PET-CT findings and response to MKI treatment in RAIR DTC patients is in line with previous reports by Marotta and cols. ( 14 ). In their work, baseline average SUVmax was significantly higher in patients who showed disease progression compared with responding subjects, however no significant correlation with PFS was found. Kim and cols. more recently described that the presence of FDG-PET uptake did not affect PFS in his cohort ( 21 ). We believe the use of PET-CT in MKI treated RAIR DTC patients should be further analyzed in larger cohorts since we found it as useful in clinical practice.

Regarding safety, most patients presented side effects during MKI treatment. Similar to previous trials, the majority of AE were low grade ( 11 , 12 ). However, in 50% of cases, reducing medication dosage was necessary at some point to manage side effects, similarly to Santos and cols. and Balmelli and cols. ( 30 , 31 ). In 25% of cases the drug was eventually suspended due to AE, also reported by Kim and cols.'s (23% permanent discontinuation) – but higher than reported by Jerkovich and cols. and Benekli and cols. with only 1 patient in each series permanently suspending sorafenib ( 18 , 21 , 25 ). Only one secondary cutaneous neoplasia was found in our cohort. Squamous cell carcinoma was found in 7 out of 207 sorafenib treated patients in DECISION trial, and other colleagues reported similar occurrences ( 11 , 13 , 18 ). The fact that almost every patient will experience AE at one point during MKI treatment and that AE might interfere with ongoing treatment highlights the importance of an experienced assistant team to manage such drugs.

Our work, however, has limitations. As a retrospective cohort, we had some cases of loss of follow-up. In addition, when we perform chart analysis, we have come across some missing data. The limited size of our sample may limit conclusions and reduce statistical power. As any study in a real-life setting, there are often difficulties in scheduling and performing exams, poor adherence to treatment, missed appointments and other factors that may interfere in some way with the results.

Nevertheless, is the first Brazilian report and one of the few subcontinental cohorts validating findings in other populations and demonstrating safety and efficacy of the use of MKI in RAIR-DTC. Our findings also corroborate previous authors that found presence of lung-only metastasis, absence of FDG uptake on target lesions on PET-CT, lower maximum SUV on PET-CT and lower Tg during treatment associated with better outcomes in RAIR DTC patients treated with MKI.

In conclusion, our analysis demonstrates that the use of MKI drugs in patients with advanced RAIR DTC is a safe and effective therapeutic approach and results were consistent with international literature data, with median PFS of 24 months (10.2-37.7) and 72.7% clinical benefit from MKI treatment. We were able to identify absence of FDG uptake on target lesions on PET-CT, lower maximum SUV on PET-CT, presence of lung-only metastasis and lower Tg during treatment as possible prognostic markers.
